# 871. Clinical Detection of Intestinal Protozoa in Digitized Modified Trichrome-Stained Stool Specimens using a Deep Convolutional Neural Network Assisted Workflow

**DOI:** 10.1093/ofid/ofad500.916

**Published:** 2023-11-27

**Authors:** Heather Rose Morris, Bobbi S Pritt, Andrew Norgan

**Affiliations:** Mayo Clinic, Rochester, Minnesota; Mayo Clinic, Rochester, Minnesota; Mayo Clinic, Rochester, Minnesota

## Abstract

**Background:**

Deep convolutional neural networks (DNN) have been successfully applied to the detection and segmentation of objects in gigapixel digital slides. In this study, trained laboratory technologists utilized a DNN-assisted digital workflow (Techcyte, Inc.; Orem, UT) to evaluate clinical stool specimens for the presence and identification of protozoan parasites.

**Methods:**

Slides were prepared from 142 stool specimens by spreading a thin layer of concentrated stool onto a slide, staining with modified-trichrome, and coverslipping with permanent mounting medium. Slides were then scanned using a NanoZoomer 360 digital slide scanner (Hamamatsu Photonics K.K., Japan) at 40x apparent magnification and prospectively analyzed by the Techcyte DNN. Objects of interest were identified and classified by the DNN (protozoa, leukocytes, erythrocytes) and presented to a laboratory technologist for interpretation. (See example image below.) DNN-assisted results were compared to those from conventional microscopic examination. To determine analytical sensitivity, slides were prepared from serial, 2-fold dilutions of 3 unique stool specimens (6 slides at each dilution/specimen) and analyzed by the DNN-assisted method and conventional microscopy.

Example of Techcyte DNN analysis review screen

**Figure:**
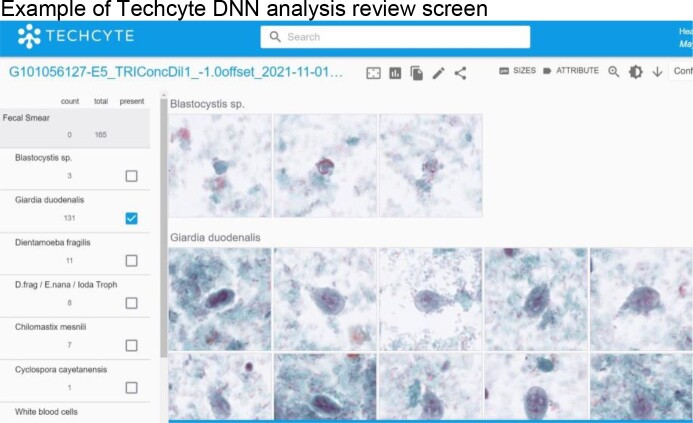

Objects of interest most likely to be significant are presented to the technologist for review in an intuitive web-based format. Parasites are confirmed as present or absent by a trained technologist after reviewing these images.

**Results:**

Digital analysis was highly reproducible, showing 100% slide level agreement (positive/negative) within and between runs using positive and negative slides. There was 98.7% and 98.3% positive and negative agreement, respectively, between conventional and DNN-assisted results. Of the 142 slides conventional microscopy detected parasites in 82, while the DNN-assisted method detected 83. The DNN-assisted method also detected more slides with leukocytes (29 vs 24) and erythrocytes (12 vs 8). All discrepant results were confirmed by repeat conventional microscopy. Analytical sensitivity of the DNN-assisted method was equivalent or superior to conventional microscopy review, detecting down to 1:64 dilutions in 6/6 slides per specimen (conventional = 1:32 to 1:64), extending for some slides to detection at dilutions of 1:2048.

**Conclusion:**

The DNN-assisted method facilitates rapid screening of negative specimens, while providing sensitive and accurate detection and classification of protozoa.

**Disclosures:**

**All Authors**: No reported disclosures

